# Clinical manifestations and long-term symptoms associated with SARS-CoV-2 omicron infection in children aged 0–17 years in Beijing: a single-center study

**DOI:** 10.3389/fped.2024.1332020

**Published:** 2024-05-15

**Authors:** Jing Li, Jingjing Li, Ling Cao, Lin Wang, Xiaobo Chen, Wenquan Niu, Li Dang, Shuzhi Dai, Ying Wang, Menglei Ge, Weijie Liu, Qinwei Song, Wenjian Xu, Lijuan Ma

**Affiliations:** ^1^ Department of Clinical Laboratory, Children’s Hospital, Capital Institute of Pediatrics, Beijing, China; ^2^ Department of Respiratory, Children’s Hospital, Capital Institute of Pediatrics, Beijing, China; ^3^ Department of Child Health Care, Children’s Hospital, Capital Institute of Pediatrics, Beijing, China; ^4^ Department of Endocrinology, Children’s Hospital, Capital Institute of Pediatrics, Beijing, China; ^5^ Center for Evidence-Based Medicine, Capital Institute of Pediatrics, Beijing, China; ^6^ Department of Outpatient Treatment Center, Children’s Hospital, Capital Institute of Pediatrics, Beijing, China

**Keywords:** SARS-CoV-2 infection, children, symptoms, long-term COVID, vaccination

## Abstract

**Objective:**

The study aims to analyze the clinical characteristics of acute phase of SARS-CoV-2 infection in children aged 0–17 years with the Omicron variant, and summarize the persistent symptoms or new-onset clinical manifestations from 4 to 12 weeks after acute COVID. Explore the association between the vaccination status and SARS-CoV-2 neutralizing antibody levels post infection among preschool-aged children. The comprehensive study systematically describes the clinical characteristics of children infected with SARS-CoV-2, providing a foundation for diagnosis and evaluating long-term COVID in pediatric populations.

**Methods:**

The study enrolled children who were referred to the Children's Hospital, Capital Institute of Pediatrics, (Beijing, China) from January 10, 2023 to March 31, 2023. Participants were classified as infant and toddlers, preschool, school-age, and adolescent groups. Children or their legal guardians completed survey questionnaires to provide information of previous SARS-CoV-2 infection history, as well as clinical presentation during the acute phase and long-term symptoms from 4 to 12 weeks following infection. Furthermore, serum samples were collected from children with confirmed history of SARS-CoV-2 infection for serological testing of neutralizing antibodies.

**Results:**

The study recruited a total of 2,001 children aged 0–17 years who had previously tested positive for SARS-CoV-2 through nucleic acid or antigen testing. Fever emerged as the predominant clinical manifestation in 1,902 (95.1%) individuals with body temperature ranging from 37.3 to 40.0°C. Respiratory symptoms were identified as secondary clinical manifestations, with cough being the most common symptom in 777 (38.8%) children, followed by sore throat (22.1%), nasal congestion (17.8%), and runnning nose (17.2%). Fatigue (21.6%), headache (19.8%) and muscle-joint pain (13.5%) were frequently reported systemic symptoms in children. The proportion of children with symptoms of SARS-CoV-2 infection varied across age groups. 1,100 (55.0%) children experienced persistent symptoms from 4 to 12 weeks post the acute phase of infection. Trouble concentrating (22.1%), cough (22.1%), and fatigue (12.1%) were frequently reported across age groups in the extended period. A limited number of children exhibited cardiovascular symptoms with chest tightness, tachycardia, and chest pain reported by 3.5%, 2.5%, and 1.8% of children, respectively. Among 472 children aged 3–5 years, 208 children had received two doses of SARS-CoV-2 vaccine at least 6 months prior to infection, and no association was found between the incidence of long-term COVID and pre-infection vaccination statuses among the 3–5 years age groups (*χ*^2^ = 1.136, *P* = 0.286).

**Conclusions:**

In children aged 0–17 years infected with SARS-CoV-2 Omicron variant, fever was the primary clinical manifestation in the acute phase, followed by respiratory symptoms, systemic non-specific and digestive presentations. In particular, respiratory and digestive system symptoms were more frequent in children aged above 6 years. Regarding the long-term symptoms from 4 to 12 weeks post-infection, the most common presentations were concentrating difficulty, cough, and fatigue. The incidence of persistent symptoms of SARS-CoV-2 did not exhibit a significant correlation with vaccination status, which was attributed to the waning efficacy of the vaccine-induced humoral immune response after 6 months.

## Introduction

The Omicron variant, designated as B.1.529, emerged in Botswana and South Africa in early November 2021. This variant has elicited significant global public health concern due to its demonstrably heightened transmissibility compared to the Delta variant and has rapidly become the prevailing circulating strain during the ongoing COVID-19 pandemic (1).

Owing to the comprehensive epidemic prevention and control strategies enacted by the Chinese government since 2020, Beijing had successfully averted any large-scale SARS-CoV-2 outbreaks until December 2022. However, following a revision of public health measures on December 7, 2022, mainland China witnessed a notable surge in SARS-CoV-2 infection, with variants BA.5.2 and BF.7 emerging as the predominant strains within the Chinese population (2). The Omicron variant has the capacity to infect most children under 17 years. Children infected SARS-CoV-2 may either remain asymptomatic or exhibit mild upper respiratory symptoms, such as fever, cough, sore throat, nasal congestion, and runny nose. Older children and adolescents might experience systemic symptoms (e.g., fatigue and headaches). Some cases may also involve gastrointestinal symptoms, including loss of taste or smell, as well as nausea and vomiting. Notably, most children with SARS-CoV-2 infection display mild symptoms and a promising prognosis, typically recovering within a span of 1 to 2 weeks (3, 4).

Several investigations have reported clinical manifestations in children infected with Omicron variant, in terms of the number of cases covered and the range of ages were limited (5, 6). Some studies have concentrated on the persistence of COVID-19 symptoms in adults, and it is increasingly evident that children also face the consequences after being infected with SARS-CoV-2 (7). However, research on prolonged symptoms in children remains limited, particularly due to small sample sizes and underrepresentation of infants and toddlers. The National Institute for Health and Care Excellence (NICE) characterizes long-COVID as the persistence or development of signs and symptoms following acute COVID-19 while remaining unattributed to alternative diagnoses, which encompasses symptomatic COVID-19 lasting from 4 to 12 weeks (8). The National Institutes of Health defines long-COVID as persistent symptoms exceeding 4 weeks after SARS-CoV-2 infection (9). This study focuses on prolonged symptoms occurring from 4 to 12 weeks after the onset of the acute phase, scrutinizing the domain of long-term COVID effects in pediatric populations meeting NICE's definition.

In the current research, a cohort of children spanning from 0 to 17 years of age, who sought care at the outpatient department of Children's Hospital, Capital Institute of Pediatrics from January 1, 2023, to March 31, 2023, were involved in the study. These young participants were thoughtfully surveyed through a meticulously designed questionnaire aimed at probing any prior SARS-CoV-2 infection. To bolster the exploration, serum samples from these children were collected for supplementary analyses, with the guardian’s informed consent, for the purpose of detecting SARS-CoV-2 neutralizing antibodies. The overarching objective of this study is to unravel the multifaceted clinical features and enduring symptoms exhibited by children of varying age groups who have been infected with the Omicron variant. Furthermore, it was attempted to explore the interplay between the levels of SARS-CoV-2 neutralizing antibodies and the persistent symptoms characterizing the long COVID. It is essential to underscore that the research was approved by the ethical approval from the Ethics Committee of the Capital Institute of Pediatrics, as authorized by the reference number of SHERLL2023016.

## Methods

### Study design and participants’ enrollment

The study was a single-center, observational real-world study conducted at the Children’s Hospital, Capital Institute of Pediatrics, which receives approximately 2.5 million annual visits. The survey questionnaires were self-reported by children (aged 8–17 years) or their legal guardians (aged 0–7 years), encompassing inquiries regarding participants’ history of SARS-CoV-2 infection and vaccination records. Confirmation of previous infections relied on positive results obtained from SARS-CoV-2 nucleic acid or antigen tests. The clinical presentation section of the questionnaire covered various aspects, including the severity of fever, respiratory symptoms (e.g., cough, sore throat, hoarseness, nasal congestion, and running nose), gastrointestinal symptoms (e.g., loss of appetite, diarrhea, vomiting, and changes in smell or taste), cardiovascular symptoms (e.g., chest tightness, chest pain, and palpitations), non-specific systemic symptoms (e.g., fatigue, headache, and muscle joint pain), as well as severe manifestations (e.g., convulsions, somnolence, and dyspnea). Furthermore, a comprehensive evaluation was conducted to identify any persistent lingering symptoms occurring from 4 to 12 weeks following SARS-CoV-2 infection. The investigation into vaccination status targeted multiple aspects, encompassing the count of vaccine doses administered, the specific vaccine's manufacturer, and the duration between the administration of the last vaccine dose and the onset of infection.

### Laboratory assays

#### Sample collection

To enhance the scientific reliability of the investigation, approximately 3 ml of blood was collected from eligible participants via venipuncture. Subsequently, serum samples were isolated by centrifugation at a speed of 3,000 rpm for a duration of 10 min and then stored at an ultra-low temperature of −80°C.

#### Quantification of SARS-CoV-2 neutralizing antibodies

The Maccura i1000 automatic chemiluminescence analyzer, manufactured by Maccura Biotechnology Co., Ltd., situated in Chengdu, China, was utilized for the assessment of SARS-CoV-2 neutralizing antibodies using a compatible reagent. The analytical procedure was designed to identify neutralizing antibodies that inhibit the interaction between the RBD domain of SARS-CoV-2 and the human ACE2 receptor. Initially, the specimen was incubated with pre-coated magnetic particles to allow the neutralizing antibodies within the specimen to bind to the RBD domain on these particles. Subsequently, a labeled form of hACE2 containing acridinium ester was introduced into the mixture, facilitating specific binding with the pre-coated magnetic particles at unoccupied RBD binding sites due to neutralizing antibody presence. The assay results were quantitatively expressed and recorded as AU/ml. A level below 6.00 AU/ml indicated a negative result, while a level equal to or higher than 6.00 AU/ml signified a positive result. This range, spanning from 3.00 to 120.00 AU/ml, was determined based on the lower limit of detection and the maximum point of the standard curve utilized in the analysis.

### Statistical analysis

The symptoms of the acute phase and long-term period were assessed as categorical variables, presented as counts and percentages, with differences among various age groups analyzed using the *χ*^2^ test. Comparison of neutralizing antibody levels between vaccinated and unvaccinated prior infection groups was conducted using the Mann–Whitney *U* test. Statistical significance was determined with a two-tailed *P*-value threshold of less than 0.05. Statistical analysis was performed via SPSS 25.0 software, which was developed by IBM Corporation. Graphical representations were meticulously crafted using GraphPad Prism 8.0 software, a distinguished product of GraphPad Software Inc., which headquartered in San Diego, CA, USA.

## Results

### Participant’s characteristics

**Figure 1:**
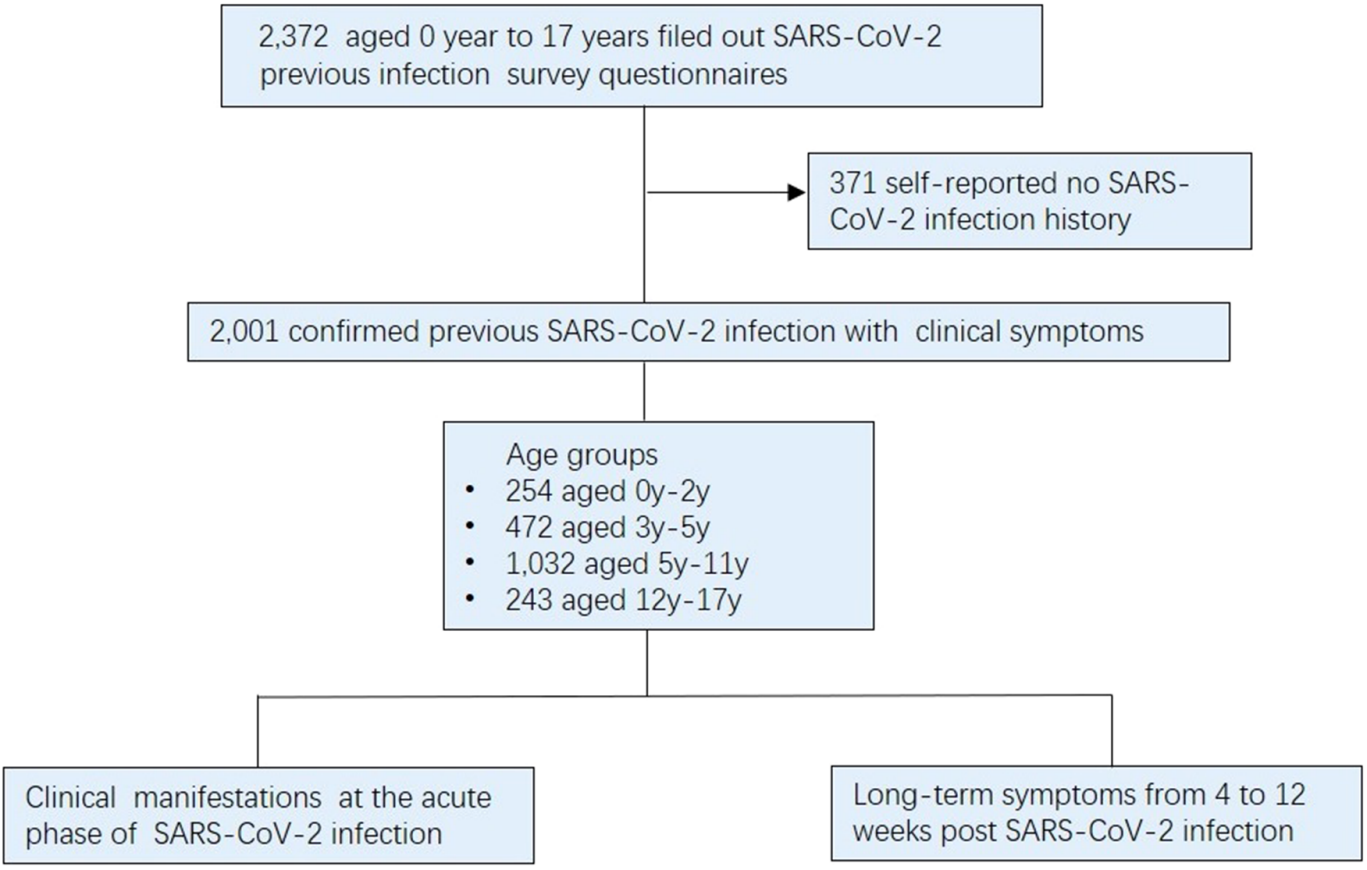
Participants’ recruitment.

During the period from January 10 to March 31, 2023, a total of 2,372 pediatric patients completed questionnaires pertaining to their SARS-CoV-2 infection history at the clinic department of Children's Hospital, Capital Institute of Pediatrics. During the survey filling process, a professional is responsible for answering questions encountered by the child or parent during the questionnaire filling process to ensure the authenticity and reliability of the questionnaire. The study's eligibility criteria excluded cases who reported no history of SARS-CoV-2-infection and didn't exhibit symptoms associated with SARS-CoV-2 infection. (*n* = 371). As a result, a final cohort consisting of 2,001 symptomatic children confirmed previously infected with SARS-CoV-2, comprising 937 boys and 1,064 girls. These participants were classified into four distinct groups based on ages: infants and toddlers (0–2 years), preschool-age (3–5 years), school-age (6–11 years), and adolescents (12–17 years) (10) (Figure 1). This study meticulously analyzed the clinical manifestations observed during the acute phase of infection and the persistence of symptoms experienced from 4 to 12 weeks post infection. It is noteworthy that most prior SARS-CoV-2 infections in children transpired in December 2022, with infection rates during early, mid, and late December being 29.7% (594/2001), 45.0% (901/2001), and 18.9% (379/2001), respectively. Furthermore, a relatively small proportion (6.4%, 127/2001) of infections occurred in early January 2023.

### Clinical manifestations in children at acute phase of SARS-CoV-2 infection

In the 2,001 children with confirmed previous SARS-CoV-2-infection, an overwhelming 95.1% of them reported experiencing fever during the acute phase. Within this group, moderate fever, characterized by a temperature range of 38.1–39.0°C, was noted in 45.6% of cases, while high fever (39.1–40.0°C) and low fever (37.3–38.0°C) were identified in 37.3% and 11.7% of instances, respectively. Only a small proportion of cases (0.4%) exhibited hyperpyrexia, with body temperature exceeding 40°C. Notably, there were no significant variations in the degree of fever across different age groups. Apart from fever, respiratory symptoms emerged as the predominant clinical manifestations in children infected with SARS-CoV-2. In the overall cohort, cough was identified as the most frequent symptom (38.8%), followed by sore throat (22.1%), nasal congestion (17.8%), running nose (17.2%), and expectoration (10.2%). The incidence rates of these respiratory symptoms exhibited an age-dependent progression, with the highest levels observed in children over the age of 12 years. The incidence of additional respiratory symptoms, such as hoarseness (4.2%), chest distress (2.0%), and shortness of breath (1.9%), remained relatively low during the acute infectious phase. In contrast, the proportion of digestive symptoms was notably lower vs. respiratory manifestations. Loss of appetite was the most prevalent among digestive symptoms, accounting for 12.8%, followed by vomiting (6.5%), smell or taste change (3.8%), and diarrhea (3.2%). Regarding non-specific clinical manifestations during the acute phase of SARS-CoV-2 infection, 21.6% of children reported experiencing fatigue, while 19.8% reported having headaches, and 13.5% mentioned muscle and joint pain. Notably, these symptoms exhibited a higher prevalence among children aged over 6 years vs. younger age groups. Additionally, somnolence was reported in 5.1% of children during the acute phase. A small number of recipients exhibited rare symptoms associated with the cardiovascular or nervous systems. Palpitations were noted in 2.0% of children, with the highest rate (6.2%) found in children older than 12 years. Convulsive symptoms were self-reported by 21 children during the acute phase, with aged under 2 years old accounting for 50% of these cases. Concerning the treatment of convulsions linked to SARS-CoV-2 infection, 4 cases admitted hospitalization, and 7 cases were referred to the emergency department, while the remaining 12 cases received care at home (Table 1).

**Table 1:** Clinical characteristics of SARS-CoV-2 infected children.

Characteristic	Total	0 years–2 years	3 years–5 years	6 years–11 years	12 years–17 years	*χ* ^2^	*p* value
	(*n* = 2,001)	(*n* = 254)	(*n* = 472)	(*n* = 1,032)	(*n* = 243)		
Sex, no. (%)							
Male	937 (46.8)	105 (41.3)	197 (41.7)	526 (50.9)	109 (44.8)	13.91	0.003
Female	1,064 (53.2)	149 (58.7)	275 (58.3)	506 (49.1)	134 (55.2)		
Vaccination dose before infection^a^, no. (%)							
0	611 (30.5)	254 (100.0)	218 (46.2)	115 (11.1)	24 (9.9)	NA	NA
1 dose	84 (4.2)	0 (0.0)	46 (9.7)	32 (3.1)	6 (2.4)		
2 doses	1,306 (65.3)	0 (0.0)	208 (44.1)	885 (85.8)	213 (87.7)		
Last dose prior to infection months interval^b^, no. (%)							
0 month	611 (30.5)	254 (100.0)	218 (46.2)	115 (11.1)	24 (9.9)	NA	NA
3–6 months	84 (3.7)	0 (0.0)	46 (9.7)	32 (3.1)	6 (2.4)		
>6 months	1,294 (64.7)	0 (0.0)	208 (44.1)	885 (85.8)	213 (87.7)		
Fever, no. (%)							
Low fever (37.3–38.0°C)	235 (11.7)	22 (8.8)	58 (12.3)	135 (13.1)	20 (8.2)	7.139	0.06
Moderate fever(38.1–39.0°C)	913 (45.6)	120 (47.2)	213 (45.1)	473 (45.8)	107 (44.0)	0.581	0.9
High fever (39.1–40.0°C)	746 (37.3)	103 (40.6)	176 (37.3)	363 (35.2)	104 (42.8)	6.284	0.098
Superhigh fever (>40.0°C)	8 (0.4)	1 (0.4)	3 (0.6)	4 (0.4)	0 (0.0)	1.639	0.65
Respiratory tract, no. (%)							
Cough	777 (38.8)	72 (28.3)	160 (33.9)	408 (39.5)	137 (56.4)	48.31	<0.001
Sore throat	442 (22.1)	13 (5.1)	57 (12.1)	252 (24.4)	120 (49.4)	178.4	<0.001
Nasal congestion	357 (17.8)	34 (13.4)	64 (13.6)	184 (17.8)	75 (30.9)	37.46	<0.001
Running nose	344 (17.2)	39 (15.4)	82 (17.4)	164 (15.9)	59 (24.3)	10.41	0.015
Expectoration	205 (10.2)	18 (7.1)	34 (7.2)	99 (9.6)	54 (22.2)	45.89	<0.001
Hoarseness	84 (4.2)	14 (5.5)	4 (0.8)	45 (4.4)	21 (8.6)	26.32	<0.001
Chest distress	40 (2.0)	0 (0.0)	6 (1.3)	19 (1.8)	15 (6.2)	28.2	<0.001
Shortness of breath	38 (1.9)	8 (3.1)	8 (1.7)	13 (1.3)	9 (3.7)	8.75	0.032
Gastrointestinal tract, no. (%)							
Loss of appetite	257 (12.8)	24 (9.4)	48 (10.2)	138 (13.4)	47 (19.3)	15.05	0.0018
Vomit	130 (6.5)	11 (4.3)	24 (5.1)	79 (7.7)	16 (6.6)	5.793	0.122
Taste or smell change	76 (3.8)	0 (0.0)	0 (0.0)	42 (4.1)	34 (14.0)	97.98	<0.001
Diarrhea	65 (3.2)	11 (4.3)	6 (1.3)	33 (3.2)	15 (6.2)	13.44	0.0038
Other tract, no. (%)							
Cardiopalmus	40 (2.0)	0 (0.0)	6 (1.3)	19 (1.8)	15 (6.2)	28.2	<0.001
Rash	27 (1.3)	6 (2.4)	7 (1.5)	13 (1.3)	1 (0.4)	3.689	0.297
Convulsion	21 (1.1)	10 (3.9)	5 (1.1)	6 (0.6)	0 (0.0)	25.15	<0.001
Systemic, no. (%)							
Fatigue	433 (21.6)	32 (12.6)	61 (12.9)	236 (22.8)	104 (42.8)	98.47	<0.001
Headache	396 (19.8)	3 (1.2)	28 (5.9)	256 (24.8)	109 (44.9)	225.1	<0.001
Muscle joint pain	270 (13.5)	5 (2.0)	31 (6.6)	158 (15.3)	76 (31.3)	117	<0.001
Somnolence	102 (5.1)	18 (7.1)	7 (1.5)	57 (5.5)	10 (4.1)	16.17	0.001

^a^ Prior to infection, 0 dose indicate unvaccinated; 1 dose indicate received 1 dose vaccine; 2 doses indicate received 2 doses vaccine.

^b^ Months interval between the last dose received and the onset of infection.

**Figure 2:**
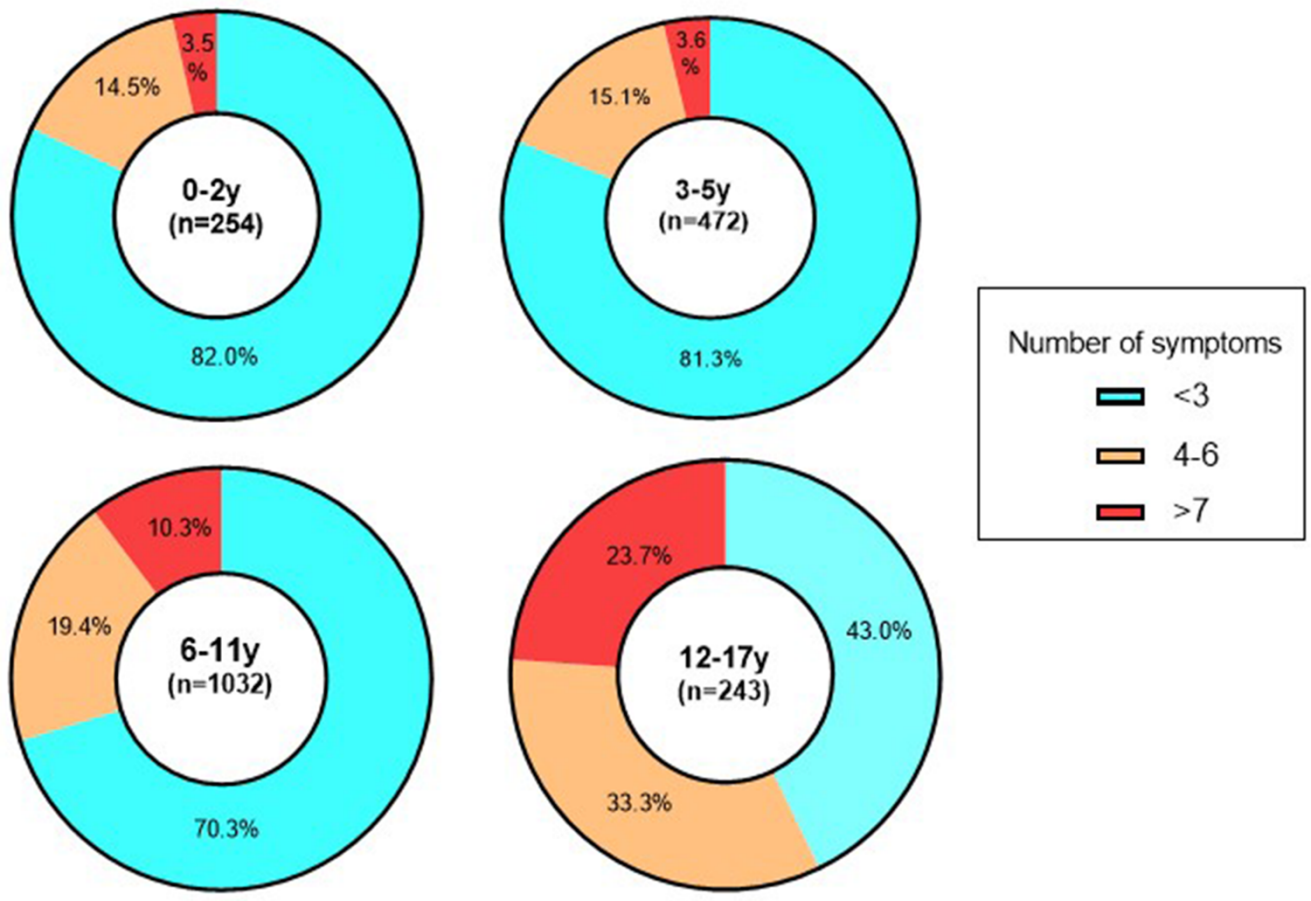
Number of symptoms in the acute phase of SARS-CoV-2 infection across age groups.

The investigation further analyzed the number of symptoms manifested by children in the acute phase of SARS-CoV-2 infection. It is noteworthy that fever was the sole symptom in 30.0% of children, encompassing moderate, high, and low fevers, which accounted for 14.7%, 10.4%, and 4.9%, respectively. Additionally, a combination of two clinical manifestations, primarily fever and cough, was observed in 9.24% of children. When exploring the association between age groups and symptomatology, a relatively majority of children aged 0–2 years (82.0%) and 3–5 years (81.3%) displayed no more than three clinical symptoms. In contrast, for children aged 6–11 years, this proportion decreased to 70.3%, whereas it further declined to 43.0% for those older than 12 years. Notably, older children demonstrated a higher burden of symptoms related to SARS-CoV-2 infection compared to their younger counterparts. Specifically, among children aged 12–17 years, a diverse range of clinical manifestations associated with SARS-CoV-2 infection was observed, with 33.3% presenting with four to six clinical symptoms and 23.7% displaying more than seven presentations. The findings underscored significant variations in the distribution of clinical symptoms across different age groups (*χ*^2 ^= 154.5, *P* < 0.001) (Figure 2).

### Long-term COVID from 4 to 12 weeks post SARS-CoV-2 infection

**Figure 3:**
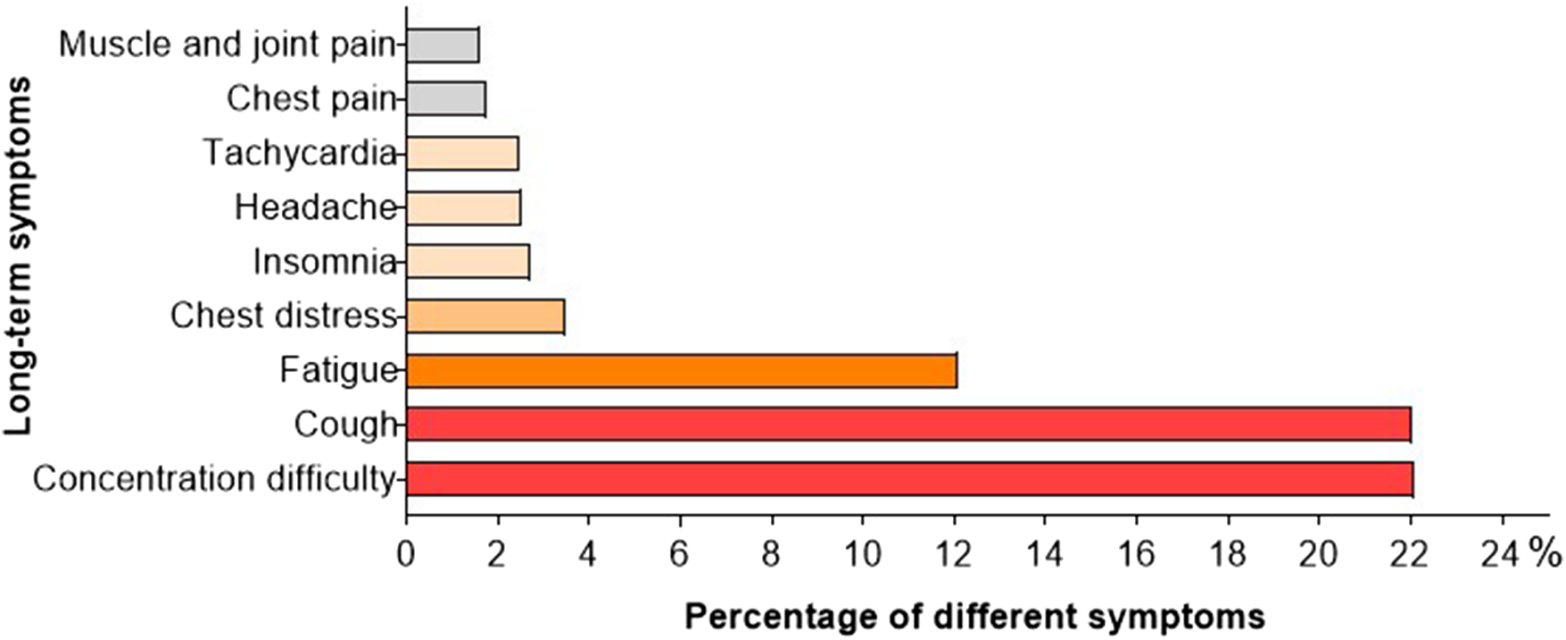
Long-term COVID from 4 to 12 weeks post SRAS-CoV-2 infection.

The primary objective of the study was to assess the clinical status of pediatric cases from 4 to 12 weeks post SARS-CoV-2 infection. Among a total of 2,001 individuals, complete recovery was observed in 45.0% (901 children), with the highest recovery rate found in the age group of 0–2 years (50.4%), followed by 3–5 years (48.9%) and 6–11 years (44.1%). The lowest rate of recovery was recorded at 35.8% for children aged 12–17 years. Conversely, 55.0% (1,101 participants) reported persistent symptoms or newly developed clinical manifestations. Difficulties in concentration were consistently mentioned across various age groups and affected 22.1% (442 children). Cough persisted in approximately the same proportion for 22.1% (442 children), while fatigue was present during an extended period for 12.1% (242 children). Regarding cardiovascular symptoms, proportions of 3.5%, 2.5%, and 1.8% experienced chest tightness, tachycardia, and chest pain respectively among children included in this study. A smaller proportion experienced insomnia (2.7%), headache (2.5%), as well as muscle and joint pain (1.6%) (Figure 3; Table 2).

**Table 2:** Long-term symptoms at 4–12 weeks following SARS-CoV-2 infection in different ages.

Characteristic, (*N* %)	Total	0 years–2 years	3 years–5 years	6 years–11 years	12 years–17 years	*χ* ^2^	*p* value
	(*n* = 2,001)	(*n* = 254)	(*n* = 472)	(*n* = 1,032)	(*n* = 243)		
Recovery	901 (45.0)	128 (50.4)	231 (48.9)	455 (44.1)	87 (35.8)	14.6	0.002
Concentration difficulty	442 (22.1)	45 (17.7)	109 (23.1)	233 (22.6)	55 (22.6)	3.283	0.35
Cough	441 (22.1)	63 (24.8)	84 (17.8)	220 (21.3)	74 (30.5)	27.19	<0.001
Fatigue	242 (12.1)	15 (5.9)	49 (10.4)	126 (12.2)	52 (21.4)	18.88	<0.001
Chest distress	70 (3.5)	0 (0.0)	10 (2.1)	44 (4.3)	16 (6.6)	20.51	<0.001
Insomnia	54 (2.7)	13 (5.1)	11 (2.3)	19 (1.8)	11 (4.5)	11.89	0.007
Headache	51 (2.5)	0 (0.0)	3 (0.6)	39 (3.6)	9 (3.7)	21.19	<0.001
Tachycardia	49 (2.5)	1 (0.4)	4 (0.8)	34 (3.3)	10 (4.1)	15.47	0.001
Chest pain	35 (1.8)	2 (0.8)	5 (1.1)	21 (2.0)	7 (2.9)	4.975	0.173
Muscle and joint pain	32 (1.6)	0 (0.0)	5 (1.1)	16 (1.6)	11 (4.5)	18.25	<0.001

Regarding diverse age groups, the most prevalent persistent symptoms among children aged 0–2 years were cough and trouble concentrating, accounting for 24.8% and 17.7%, respectively. Among children aged 3–5 and 6–11 years, trouble concentrating, cough, and fatigue were predominant, with rates ranging from 10.4% to 23.1%. In adolescents aged 12–17 years, there was a higher prevalence of cough (30.5%), difficulty in concentration (22.6%), and fatigue (20.6%) compared to younger age groups.

### Relationship between SARS-CoV-2 neutralizing antibodies and long-term COVID in preschool-aged children

In the present study, a total of 1,747 children aged 3–17 years met the age criteria for receiving inactivated SARS-CoV-2 vaccine in China. The majority of children in the age groups 6–11 and 12–17 had been immunized prior to infection, with vaccination rates of 88.8% and 90.1%, respectively. Among the preschool-aged group (3–5 years) consisting of 472 children, there were 218 individuals who remained unvaccinated, while 254 had received at least one dose of the vaccine prior to infection. Within the vaccinated group of preschool-aged children, a subset of 208 individuals had received primary immunization of two doses vaccine, with the last dose administered more than six months prior to SARS-CoV-2 infection. An analysis was conducted to explore the association between pre-infection SARS-CoV-2 vaccination status and the presence of long-term symptoms in infected children aged 3–5 years. Among those who had received two vaccine doses prior to infection (*n* = 208), complete recovery was achieved by 118 individuals; similarly, in the unvaccinated group (*n* = 218), recovery was observed in 112 children as well. No significant differences were found in the incidence of long-term COVID among preschool-aged children with different vaccination statuses (*χ*^2^ = 1.136, *P* = 0.286).

**Figure 4:**
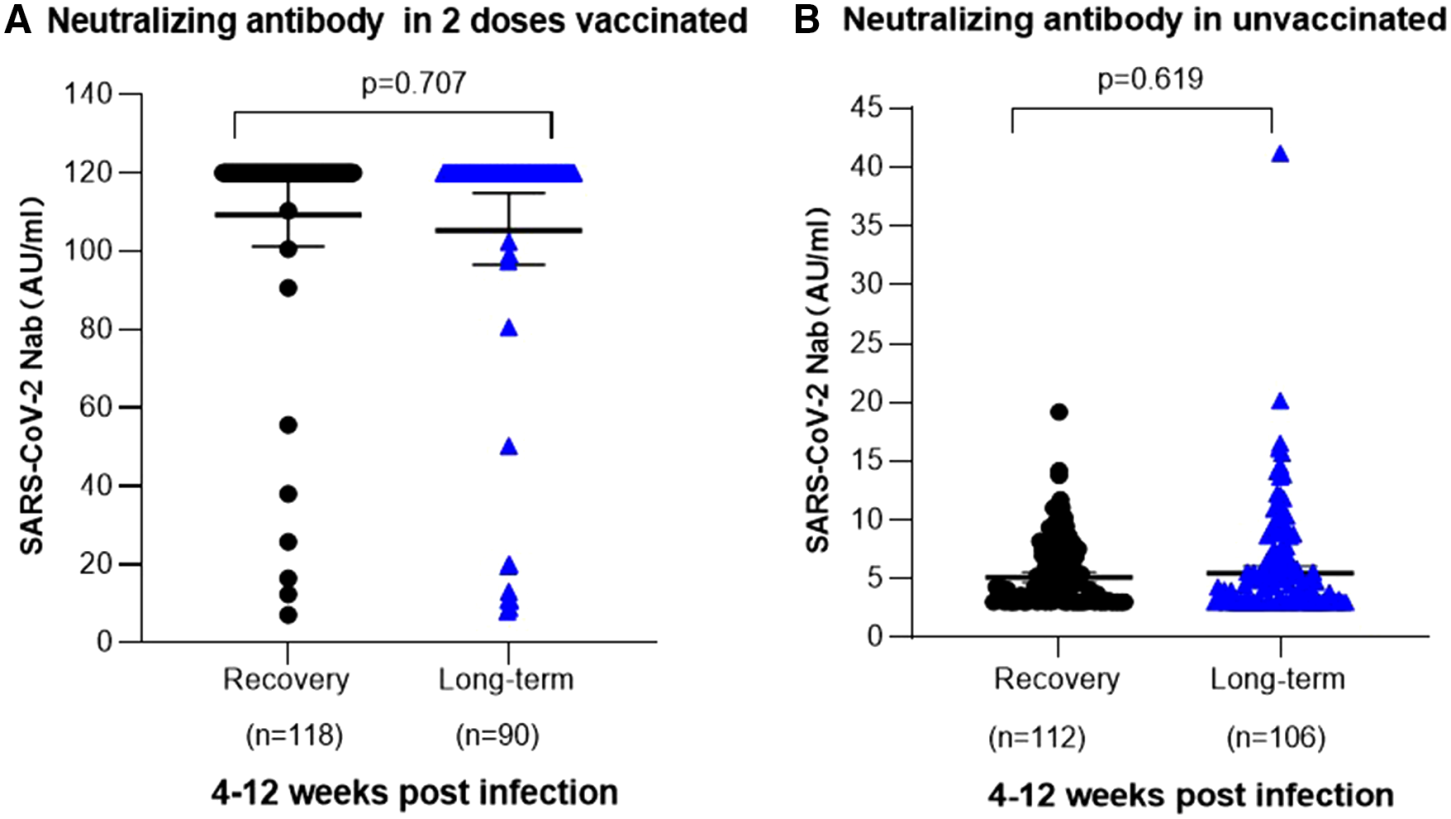
SARS-CoV-2 neutralizing antibodies in 3-5 years old children post infection. (**A**) Relationship between the occurrence of long-term symptoms of SARS-CoV-2 infection and the levels of neutralizing antibodies in children aged 3–5 years who received two doses of vaccination prior to infection. Among 118 children who were fully recovered, the mean neutralizing antibody titer was 114.70 AU/ml (95% Cl 101.20–117.90), whereas among 136 children with long-term symptoms, it was [113.20 AU/ml (95% Cl 96.49–114.90)]. No significant difference in neutralizing antibody level was noted in different clinical outcomes (*P* = 0.707). (**B**) Association between the occurrence of prolonged symptoms following SARS-CoV-2 infection and the levels of neutralizing antibodies in unvaccinated children aged 3-5 years. 112 children achieved complete recovery, with a mean neutralizing antibody level of 5.73 AU/ml (95% CI, 5.17–6.28). Among 106 children experiencing persistent symptoms, the mean neutralizing antibody level was 6.51 AU/ml (95% CI, 5.55–7.47). No significant difference was identified in neutralizing antibody level of different clinical outcomes (*P* = 0.619).

Moreover, a further analysis scrutinized the post-infection levels of SARS-CoV-2 neutralizing antibodies and their correlation with persistent symptoms. Among children aged 3–5 years, vaccinated individuals consistently exhibited elevated levels of SARS-CoV-2 neutralizing antibodies, regardless of whether they experienced persistent symptoms. The measurements of SARS-CoV-2 neutralizing antibodies showed values of 114.70 AU/ml (95% CI, 101.20–117.90) for fully recovered children and 113.20 AU/ml (95% CI, 96.49–114.90) for children with prolonged COVID-19 symptoms. Notably, no significant difference was achieved in SARS-CoV-2 antibody levels between fully recovered children and those with persistent symptoms in the vaccinated group (*P* = 0.707). For non-vaccinated children prior to infection, both fully recovered individuals and those with prolonged symptoms had relatively low levels of SARS-CoV-2 neutralizing antibodies at 5.73 AU/ml (95% CI: 5.17–6.28) and 6.51 AU/ml (95% CI: 5.55–7.47), respectively. Moreover, no statistically significant difference was observed in SARS-CoV-2 neutralizing antibody levels between fully recovered individuals and those with persistent symptoms within the unvaccinated group (*P* = 0.619). (Figures 4A,B).

## Discussion

The emergence of the Omicron variant, characterized by its heightened transmissibility compared to the Delta variant, has led to a substantial surge in SARS-CoV-2 infection within the pediatric population (11–13). Since December 2022, the SARS-CoV-2 Omicron variant, characterized prominently by the BA.5.2 and BF.7 strains, has been circulating in China. Notably, children spanning various age groups have exhibited a heightened susceptibility to the Omicron variants. This study emphasized fever as the primary clinical manifestation of SARS-CoV-2 Omicron variant infections in children across all ages, with approximately half of them presenting body temperatures ranging from 38.1 to 39.0°C. Respiratory symptoms emerged as the second most frequent category, with cough being the predominant clinical feature in over one-third of the cases. Supplementary respiratory symptoms, comprising conditions (e.g., sore throat, nasal congestion, and runny nose), were manifested with varying incidence rates falling within the range of 17.2%–22.1%. Notably, these respiratory symptoms were more prevalent in children aged 6 years and older. The study also revealed a noteworthy age-related increase in the prevalence of sore throats, with approximately half of children aged 12 years and above reporting the symptom during the acute phase. Regarding gastrointestinal symptoms, loss of appetite emerged as the most reported manifestation. Systemic clinical presentations, such as fatigue, headache, and muscle and joint pain, were frequently observed among children older than 6 years. Tachycardia was identified in a limited number of older children. Most participants experienced a mild course of illness and did not seek medical attention at a hospital emergency department during the acute phase of infection. However, convulsive symptoms were reported by 21 children, all of whom presented with a high fever ranging from 39.1 to 40.0°C. In terms of treatment, hospitalization was required for 4 children during the acute phase of infection.

Several studies have described the clinical presentation during the acute phase of SARS-CoV-2 infection in pediatric populations. A Canadian study investigated 1,440 SARS-CoV-2-infected children across different COVID-19 variants uncovered that fever and cough were more prevalent in cases associated with Omicron variants in comparison to both the original virus and the alpha variant. These symptoms affected 81% and 58.8% of the 468 children with the Omicron variant, respectively (14). As the highly contagious Omicron variant continues to spread, there has been a continuous rise in pediatric SARS-CoV-2 infections. Another study conducted in South Africa revealed that the number of pediatric hospitalizations among those aged 19 years or younger exceeded previous SARS-CoV-2 pandemic waves. Among these instances, fever was the predominant symptom in 61% of cases, followed closely by cough, which presented in 57% of cases. Additionally, symptoms (e.g., shortness of breath, seizures, vomiting, and diarrhea) were noted (5, 15). In our study, it revealed a progressive increase in the number of symptoms during the acute phase of COVID-19 infection with advancing age among children. Some studies compared the symptoms burden in the Omicron-dominant period, which unveiled children in the 0–4 age group exhibited fewer symptoms vs. those aged 5–17 years at the acute phase (16). The adolescent (aged 12–17 years) demonstrated a higher number of symptoms than children (aged 0–11 years) (17). In terms of specific clinical symptoms, children between the ages of 5–17 years had higher rates of headache, muscle pain, fatigue, sore throat, and alterations in taste or smell vs. children who were aged 0–4 years (15, 18). These findings align with results of the current investigation. The current study has revealed a significant decrease in the occurrence of cough, sore throat, and expectoration among younger children with respiratory symptoms during the acute phase of SARS-CoV-2 infection compared to their older counterparts, potentially attributed to the underlying factor. The production of type I IFNs by respiratory epithelial cells is essential for host defense against SARS-CoV-2, which depends on the age. The mucous membranes of young children exhibit a propensity for type 1 interferon responses, which play a crucial role in facilitating local defenses against SARS-CoV-2 infection (19, 20).

An analysis of clinical symptoms in adult patients with mild Omicron variant infection in China revealed that throat symptoms were the most common (49.12%), followed by nasal symptoms (20.08%). Additionally, 55.26% of adults with mild Omicron variant infections experienced general systemic symptoms (21). In the context of this ongoing investigation, it is notable that the occurrence of upper respiratory symptoms, encompassing several manifestations (e.g., sore throat, nasal congestion, and runny nose), in children aged 12 years and above who have contracted the Omicron variant was strikingly analogous to that in adults. Furthermore, the prevalence of non-specific symptoms (e.g., headache and fatigue) among older children bears a strong resemblance to the patterns seen in adults.

Currently, a substantial segment of the pediatric population is confronted with the potential of enduring prolonged consequences following their exposure to SARS-CoV-2 infection. The World Health Organization (WHO) has operationalized the definition of “long COVID” as the persistence of symptoms for a minimum duration of 2 months, occurring three months after the onset of COVID-19, significantly impairing an individual's daily life and can't be explained by other alternative diagnosis (22). However, another guideline suggests that symptoms lasting for 3 months or longer post-infection (23).

A recent study demonstrated that vaccinated children who had previously experienced Omicron infection exhibited a 94.3% protection rate against re-infection within three months, and a 79.4% protection rate within four months post-vaccination (24). According to the observations in this study, reinfection has already occurred in a portion of the population by April 2023 (i.e., four months after initial infection). Additionally, in the Beijing area, the influenza A virus has been circulating during spring 2023, and children with influenza also showed symptoms similar to those of Omicron infection. To ensure a clear background of a single viral infection, accurately assess the persistent symptoms of initial infection with Omicron, and avoid the impact of additional viral infections on the evaluation of clinical symptoms, this study analyzed the clinical symptoms of children who had been previously infected with Omicron variant infection from 4 to 12 weeks after initial exposure.

Overall, recovery was observed in 45.0% of participants, with the highest rates observed among children aged 0–2 years. The prevalence of long COVID demonstrates a positive correlation with the age of children, peaking among adolescent individuals. A prospective study investigating risk factors for long COVID in children revealed that those aged 11 years and above were associated with the development of post-COVID-19 symptoms starting from three months after infection (25). Trouble concentrating, cough, and fatigue were identified as the three predominant persistent symptoms following Omicron infection. Furthermore, the prevalence of long-term symptoms varied across different age groups and was more common in children over 6 years old. In addition, a limited number of children experienced cardiovascular discomfort, reporting symptoms, such as chest tightness, tachycardia, and chest pain. Recent studies have indicated that approximately 60% of cases with COVID-19 experience long-term symptoms within the first year after the acute phase of the disease. A review summarized a long-term follow-up of adults after coronavirus infection, regardless of the specific virus variant. Fatigue was the most prevalent long-term COVID-19 symptom, followed by pain and cough (26). An additional meta-analysis unveiled that within the demographic of cases with symptoms that endure beyond a span of 4 weeks post-SARS-CoV-2 infection, the prevalence of long COVID in children and adolescents amounted to approximately 25.24%. The most recurrently reported manifestations encompassed mood-related symptoms, persistent fatigue, sleep disturbances, recurring headaches (7). A recent study investigated long-term COVID symptoms over 2 months in children aged 0–14 years with a confirmed history of SARS-CoV-2 infection compared to matched controls. The findings demonstrated that 26.5%–32.3% of children infected with SARS-CoV-2 developed long-term COVID-19, with mood swings, coughing, and rashes common in younger children, while trouble concentrating and fatigue were common in school-aged participants. A number of investigators have documented elevated rates of persistent symptoms, notably mood fluctuations, among cases who were not infected with SARS-CoV-2 vs. children who were afflicted, indicating the potential influence of the pandemic period’s social restrictions and resultant psychological ramifications (27, 28).

In the present investigation, approximately half of the preschool-aged children had received two doses of the SARS-CoV-2 vaccine, with the majority receiving the second dose at least six months prior to infection. As vaccination time passed by, the humoral immune response-induced antibody production declined to levels similar to baseline at the time of infection (29, 30). The current study revealed no discernible association between vaccination status and the incidence of persistent symptoms in children. However, a correlation was observed between pre-infection vaccination status and levels of SARS-CoV-2 neutralizing antibodies in children. Those who were vaccinated exhibited higher levels of neutralizing antibodies irrespective of experiencing persistent symptoms. In contrast, both fully recovered children and those with persistent symptoms in the unvaccinated group demonstrated relatively low or even negative antibody levels.

The benefits of SARS-CoV-2 vaccine have been approved. A study conducted during the Omicron period in Chile estimated the effectiveness of the CoronaVac vaccine in children aged 3–5 years. The results demonstrated its efficacy in preventing symptomatic infection, hospitalization, and ICU admission at a rate of 38.2%, 64.6% and 69.0% respectively (31). While vaccination reduced the risk of severe COVID-19 infection, ongoing researches are currently investigating the impact of pre-infection vaccination on the development of long COVID in both adult and pediatric populations. It is crucial to emphasize that the effect of vaccination on long COVID may vary across studies and can be influenced by factors such as timing of vaccination and study methodology. A study indicated that prior vaccination did not provide protection against several long COVID symptoms in adults (32). Nonetheless, specific studies have suggested a reduced risk of experiencing protracted long COVID symptoms in adults who have undergone vaccinations (33). In term of pediatric long COVID research, a study found no significant difference in persistent symptoms occurrence three months post delta variant infection between vaccinated adolescents and their unvaccinated counterparts (34). Nevertheless, a recent analysis examined the prevalence of persistent symptoms among different age groups of children based on the pre-infection vaccination status. Although vaccinated individuals had a small decreased risk of post-COVID-condition, no statistical significance was observed between primary vaccinated and unvaccinated children (25). In this study, most preschool children who received vaccinations completed their primary immunization at least 6 months prior to contracting COVID-19. It was observed that the efficacy of the vaccine-induced humoral immune response waned after a period of 4–6 months. Consequently, upon exposure to SARS-CoV-2, protection levels gradually decline towards their pre-vaccination status.

The study delved into the clinical manifestations in children across a wide age spectrum, encompassing ages 0–17, with a specific focus on the age groups of 0–2 years and 3–5 years. Additionally, it analyzed the long-term symptoms associated with SARS-CoV-2 during the 4–12 weeks post-infection period in children spanning all age groups. In addition, within the cohort of children aged 3–5 years, the study investigated the impact of pre-vaccination on the risk of infection and the incidence of long-term symptoms. It also compared the levels of neutralizing antibodies in children who had fully recovered from COVID-19 and those who continued to experience persistent symptoms in pre-vaccinated and unvaccinated pediatric population. Several limitations should be acknowledged in this study. First, most symptoms of SARS-CoV-2 infection were reported by the children themselves or their guardians, expect clinical manifestations of cardiovascular system assessed by medical professionals. Second, the study did not include a control group of children, consisting of both unvaccinated and vaccinated uninfected individuals, which would have provided a more precise analysis of the long-term symptoms that occur during the 4–12 weeks following SARS-CoV-2 infection. Third, the survey questionnaire did not comprehensively cover the more prevalent emotional changes in the long-term course of COVID-19. In addition, the limited duration of follow-up hinders effective summarization of trends in long-term COVID-19 symptoms. Although the study investigated 371 children who self-reported no prior history of SARS-CoV-2 infection, it is likely that these represented asymptomatic cases when the co-occupant was a confirmed case, and they may not be suitable as a control group for further analysis.

## Conclusion

The study revealed that fever was the predominant clinical manifestation in children spanning age of 0–17 years infected with the Omicron variant. Respiratory symptoms followed, with approximately one-third of the children experiencing cough, followed by other symptoms (e.g., sore throat, nasal congestion, and runny nose). Systemic symptoms, such as fatigue, headache, and muscle-joint pain were frequently found. The proportion of SARS-CoV-2 infection symptoms displayed variations across diverse age groups. Around half of children continued to experience persistent symptoms in the 4–12 weeks following the acute phase of SARS-CoV-2 infection Trouble concentrating, cough, and fatigue were among the frequently reported symptoms, particularly in the older age groups of 6–17 years. Within the cohort of children aged 3–5 years, nearly half had received the SARS-CoV-2 vaccine at least 6 months prior to their infection. The study findings suggest that there is no apparent link between vaccination status and the incidence of persistent symptoms associated with COVID-19, which may attribute to protection provided by vaccine vaned over time. The study provided outstanding insights for diagnosing and assessing the long-term symptoms of SARS-CoV-2 in pediatric populations.

## Data Availability

The raw data supporting the conclusions of this article will be made available by the authors, without undue reservation.
